# Acute Vision Loss Following Endoscopic Sinus Surgery

**DOI:** 10.1155/2017/4935123

**Published:** 2017-02-13

**Authors:** Serena Byrd, Adnan S. Hussaini, Jastin Antisdel

**Affiliations:** Department of Otolaryngology-Head and Neck Surgery, Saint Louis University School of Medicine, Saint Louis, MO, USA

## Abstract

A 41-year-old female with a history of uterine cancer and Celiac and Raynaud's Disease presented to our institution with frequent migraines and nasal congestion. She underwent functional endoscopic sinus surgery (FESS) and experienced acute unilateral vision loss postoperatively. Rapid recognition of the etiology and effective treatment are paramount given the permanent and irreversible vision loss that can result. Arterial vasospasm following FESS is rare. Patients with autoimmune diseases have perhaps an increased risk for vasospasm secondary to an increased vasoreactive profile. We present the first documented case of nitroglycerin sublingual therapy to successfully treat ophthalmic artery vasospasm following FESS. Nitroglycerin sublingual therapy is a promising treatment for ophthalmic vasospasm secondary to its ability to cross the blood-ocular barrier, its rapid onset of action, and its ability to promote relaxation of vascular smooth muscle.

## 1. Introduction

Acute postoperative vision loss is a rare but devastating complication of functional endoscopic sinus surgery (FESS). The most feared orbital complication following routine sinus surgery is orbital hematoma. Orbital hematoma is oftentimes obvious to recognize on physical examination by rapid unilateral orbital swelling and thus is easily treated with decompression (i.e., lateral canthotomy and cantholysis). Injury to orbital structures, thromboembolic event, retinal migraine, and ophthalmic artery vasospasm are other etiologies of unilateral vision loss that are oftentimes more difficult to recognize clinically [1]. Rapid identification of the underlying etiology of vision loss is paramount because permanent and irreversible vision loss often occurs within 60–90 minutes following vascular compromise and treatment differs based on etiology.

Our patient underwent topical decongestion with oxymetazoline (Afrin). Oxymetazoline is a long-acting imidazoline derivative with an onset of action under 10 minutes and duration of action of 6 hours or longer. It acts locally as a sympathomimetic vasoconstrictor, causing shrinkage of the nasal mucosa [2, 3]. There are few documented cases of suspected retinal artery branch occlusion and segmental cerebral vasoconstriction following excessive home use of oxymetazoline [4] and no reports to suggest vasoconstriction to the ocular vasculature following endoscopic sinus surgery. However, there have been several documented cases of ophthalmic artery vasospasm following FESS thought to be secondary to direct injection of epinephrine into the ethmoidal artery [5].

This case report is unique in that it is the first documented case of acute vision loss without local injection or associated orbital hematoma and the first reported case of nitroglycerin sublingual therapy to successfully treat suspected ophthalmic artery vasospasm.

## 2. Case Presentation

**Figure 1:**
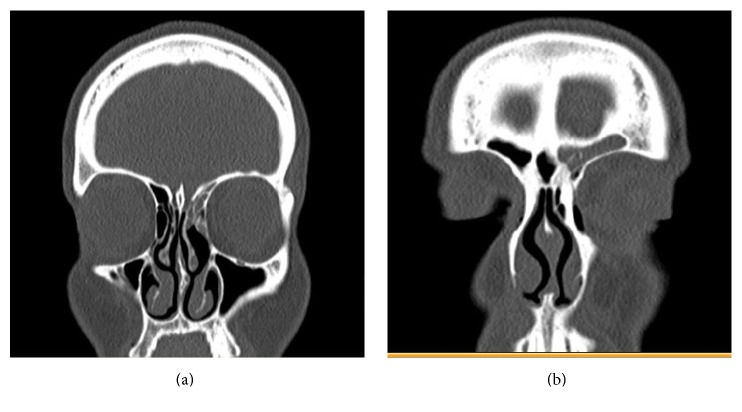
Preoperative coronal CT scan demonstrating evidence of soft tissue density consistent with known inverting papilloma in ethmoid (a) and frontal (b) sinuses.

A 41-year-old female with a history of Raynaud's disease and self-reported Celiac disease presented to our institution with frequent migraines and nasal congestion. She was found to have a recurrent inverting papilloma of the left frontal and ethmoid sinuses (Figure 1). She had previously undergone resection of this inverting papilloma at an outside hospital, which was complicated by a delayed CSF leak requiring placement of a lumbar drain. On initial presentation to our institution, her physical exam was unremarkable except for nasal endoscopy revealing a polypoid mass emanating from the left frontal sinus outflow tract.

She underwent an uncomplicated endoscopic modified Lothrop for approach and resection of the tumor. Perioperatively, topical oxymetazoline was used with no injection of local anesthetic. The patient was noted to have tumor superior to the anterior ethmoid artery and pedicled off the lamina papyracea. In order to completely remove the tumor, the anterior ethmoidal artery required ligation using bipolar followed by monopolar cautery. The superior aspect of the lamina papyracea was drilled away with only a small amount of orbital fat protruding from the foramen of the anterior ethmoidal artery. The lesion was completely removed with no significant blood loss and no CSF leak.

The patient remained hemodynamically stable throughout her postoperative course. However, approximately 12 hours after her surgery, she began to experience left sided eye pain and slight eye pressure but denied visual changes or other symptoms. This progressed over the next several hours to left sided blurry vision and prompted immediate ENT evaluation. Physical exam showed soft, moderate fullness of the left periorbital region, intact extraocular movement, and no proptosis. Ophthalmology was immediately consulted and fundoscopic exam showed pallor of the left retina and optic disk with an inferior visual loss and an afferent pupillary defect.

This was treated immediately with one nitroglycerin sublingual tablet with prompt return of her vision within several minutes of administration. Repeat fundoscopic examination after nitroglycerin treatment revealed a normal appearing retina and optic disk.

## 3. Discussion

Our case report is unique in that it is the first documented case of nitroglycerin sublingual therapy to successfully treat suspected ophthalmic artery vasospasm. Informed consent was obtained from the patient prior to writing of this manuscript.

In our patient, the etiology of her unilateral vision loss was felt to be most consistent with retinal artery vasospasm. It was felt that an orbital hematoma was unlikely given that the patients symptoms developed long after the completion of her surgery (>12–18 hours). Although the patient does have a prior history of uterine cancer, a thromboembolic event was also felt to be an unlikely etiology given that she has remained cancer free, is otherwise young and healthy, and experienced a prompt response to nitroglycerin treatment. Carotid artery disease (CAD) was also not felt to contribute as our patient had no preceding history or risk factors for such disease. Given that her symptoms did not start until more than 12 hours after surgery (at which time oxymetazoline should no longer be present) and the fact that our patient had such a rapid improvement in her symptoms and ophthalmologic exam after sublingual nitroglycerin treatment, we find it most likely that retinal vasospasm was the cause of her vision loss.

The anterior and posterior ethmoidal arteries provide a direct connection of the nasal cavity and orbit via their continuity with the ophthalmic artery. This vascular connection demonstrates how local anesthetic with epinephrine injected within the nasal cavity could cause retrograde flow, vasoconstriction to the blood supply of the optic nerve, and subsequent vision loss [5]. Although no injection of local anesthetic was performed in our patient, perhaps drilling of the lamina at the region of the ethmoid artery foramen to control bleeding could have contributed to vasospasm and compromised optic nerve perfusion. Retinal vasospasm may also occur in patients with migraines or migraine risk factors [6].

Studies have also suggested that patients with a history of migraine headaches and Raynaud's Disease, both comorbidities that our patient experienced, are more likely to suffer from vasospastic syndrome. Vasospastic syndrome is characterized by a more vasoreactive response to stimuli such as cold, emotional stress, or possibly vasoconstrictive medications. It has also been reported to occur more frequently in women of childbearing age. Some patients suffer from migraines, but the relationship between vasospastic syndrome and migraines is rather weak [7]. Choroidal and optic nerve circulation involvement in vasospastic syndrome has been previously described. While the exact pathophysiology of focal retinal vasospasm remains unknown, it has been speculated that endothelin-1 (ET-1), which increases in all diseases related to vasospasm, contributes to retinal vasoconstriction by inducing vascular hyperresponsiveness to various stimuli rather than being the direct cause of vasospasm [7].

The regulation of metabolic substrates and oxygen to the retina is a complex process due to the presence of two vascular systems, which differ anatomically and physiologically: the retinal and the choroidal systems. These systems form what is known as the blood-ocular barrier. The blood-ocular barrier is a highly selective barrier formed by tight junctions along the endothelial cells and is similar in composition to the blood-brain barrier. Physical or oxidative stress, as well as inflammatory events, may affect the permeability of the intercellular junctions. The extraocular and choroidal vessels are autonomically innervated. The retinal vessels lack autonomic supply, such that blood flow is controlled solely by local vascular control mechanisms determined by a balance of ET-1 and endothelium-derived nitric oxide (NO) [8].

Vasospasm has been described extensively in the neurosurgery and vascular literature. It is often diagnosed by a high level of clinical suspicion and can be confirmed by angiography. “Triple-H” (hypertension, hypervolemia, and hemodilution) therapy and calcium channel blockers have been shown to improve treatment outcomes. Catheter angiography with infusion of vasodilators and balloon angioplasty to mechanically open the stenosed vessel and break the vasospasm cycle have also been described. Treatment of acute monocular vision loss related to vasospasm includes prompt treatment with calcium channel blockers, which improves blood flow by reducing the vasoconstrictive effects of ET-1 [9]. If standard therapy for vasospasm fails to correct the underlying problem/symptom, typically other etiologies such as thromboembolic event, migraine, or surgical injury should be considered and further investigated [10].

In our particular case report, nitroglycerin was used for the treatment of retinal vasospasm. This drug is well known for its treatment of coronary artery spasm, angina, and myocardial infarction [11]. It often produces a severe headache shortly after administration because nitroglycerin readily crosses the blood-brain barrier and causes vasodilation. In addition, nitroglycerin offers a rapid onset of action, which is extremely important as permanent irreversible vision loss is typically seen at 60–90 minutes after vascular compromise. Given the similarities of the blood-ocular barrier to the blood-brain barrier, it seems that a rationale and effective treatment of retinal vasospasm is nitroglycerin sublingual therapy.

In summary, arterial vasospasm following FESS is rare. However, prompt recognition and effective treatment are vital in preserving vision. Patients with Raynaud's Disease and/or a history of migraines are perhaps more at risk for vasospasm secondary to a more vasoreactive profile [7]. Nitroglycerin sublingual therapy is a promising treatment for ophthalmic vasospasm secondary to its ability to cross the blood-ocular barrier, its rapid onset of action, its relative good safety profile, and its ability to promote relaxation of vascular smooth muscle. This medication, although not previously documented for the treatment of ophthalmic artery vasospasm, should be considered as an alternative treatment or in addition to standard calcium channel blockers therapy.

## References

[1] Pula J. H., Kwan K., Yuen C. A., Kattah J. C. (2016). Update on the evaluation of transient vision loss. *Clinical Ophthalmology*.

[2] Higgins T. S., Hwang P. H., Kingdom T. T., Orlandi R. R., Stammberger H., Han J. K. (2011). Systematic review of topical vasoconstrictors in endoscopic sinus surgery. *Laryngoscope*.

[3] Şahin M. İ., Kökoğlu K., Güleç Ş., Ketenci İ., Ünlü Y. (2016). Premedication methods in nasal endoscopy: A Prospective, Randomized, Double-Blind Study. *Clinical and Experimental Otorhinolaryngology*.

[4] Fivgas G. D., Newman N. J. (1999). Anterior ischemic optic neuropathy following the use of a nasal decongestant. *American Journal of Ophthalmology*.

[5] Savino P. J., Burde R. M., Mills R. P. (1990). Visual loss following intranasal anesthetic injection. *Journal of Clinical Neuro-Ophthalmology*.

[6] Evans R. W., Grosberg B. M. (2008). Retinal migraine: migraine associated with monocular visual symptoms. *Headache*.

[7] Flammer J., Pache M., Resink T. (2001). Vasospasm, its role in the pathogenesis of diseases with particular reference to the eye. *Progress in Retinal and Eye Research*.

[8] Riva C. E. (2011). Ocular circulation. *Adler's Physiology of the Eye*.

[9] Meyer P., Lang M. G., Flammer J., Lüscher T. F. (1995). Effects of calcium channel blockers on the response to endothelin-1, bradykinin and sodium nitroprusside in porcine ciliary arteries. *Experimental Eye Research*.

[10] Naylor A. R., Robinson T. G., Eveson D., Burns J. (2014). An audit of management practices in patients with suspected temporary monocular blindness. *British Journal of Ophthalmology*.

[11] Hung M.-J., Hu P., Hung M.-Y. (2014). Coronary artery spasm: review and update. *International Journal of Medical Sciences*.

